# Evaluation of Mediterranean sponges as natural samplers for environmental DNA (eDNA)

**DOI:** 10.12688/openreseurope.19755.1

**Published:** 2025-03-17

**Authors:** Nicolas Garcia-Seyda, Marielle Garcia, Dorian Guillemain, Aurélie Bonin

**Affiliations:** 1 Aix Marseille Univ, Université de Toulon, CNRS, IRD, MIO, Marseille, 13288, France; 2 Tropical Marine Ecology of Pacific and Indian Oceans - UMR 9220, Research Institute for Development (RID), Noumea, 98848, New Caledonia; 3 NGS consultancy, Marseille, 13008, France; 4 Argaly, Sainte-Hélène-du Lac, 73800, France

**Keywords:** Environmental DNA (eDNA), natural sampler DNA (nsDNA), Fish Metabarcoding, Marine sponges, Benthic biodiversity, Axinella verrucosa, Sponge-based biomonitoring

## Abstract

Marine sponges have emerged as effective natural samplers of environmental DNA (eDNA), offering a promising alternative for biodiversity monitoring. By filtering large volumes of seawater, sponges accumulate eDNA from surrounding communities, potentially enhancing species detection in marine environments where conventional water sampling is limited. In this study, we evaluated the eDNA recovery efficiency of seven Mediterranean sponge species to identify optimal candidates for biomonitoring. *Axinella verrucosa* outperformed other tested species, highlighting its potential for long-term biodiversity assessments. Our results align with previous findings that low microbial abundance (LMA) sponges recover more eDNA than high microbial abundance (HMA) species, reinforcing the need for targeted sponge selection in future studies. Detected fish taxa were all bottom dwelling, supporting the relevance of sponge eDNA for monitoring cryptic species and benthic habitats. As eDNA-based monitoring advances, sponge sampling offers a valuable complement to water eDNA surveys, particularly in habitats where conventional sampling is challenging.

## Plain Language Summary

Marine sponges naturally filter seawater and collect tiny traces of DNA (eDNA) from nearby organisms. This makes them useful for detecting marine species, especially in places where traditional water sampling is difficult. In this study, we tested seven Mediterranean species to see which ones were best at capturing eDNA. *Axinella verrucosa* performed the best, meaning it could be an option for long-term biodiversity monitoring. Our results also confirmed that sponges with fewer internal microbes (LMA sponges) collect more eDNA than those with high microbial loads (HMA sponges). Most of the fish detected were bottom-dwelling species, showing that sponge eDNA is particularly useful for studying life at the sea bottom.

## Introduction

Environmental DNA (eDNA) has emerged as a revolutionary tool for marine biodiversity monitoring, offering a valuable complement to traditional methods based on visual surveys or species capture ^1^. eDNA-based approaches often demonstrate superior sensitivity, particularly for detecting species that are rare, cryptic, small, nocturnal, or otherwise elusive, which are frequently missed by conventional techniques ^2^. A significant bottleneck in current methodologies is the time-consuming water filtration process, typically restricted to surface waters due to the logistical challenges of sampling at depth. This limitation may result in the loss of valuable information on benthic biodiversity. While technological advances, such as submersible pumps and robotic sampling devices ^3, 4^, are being developed to enable deeper sampling, they often remain prohibitively expensive. Additionally, for divers using manual underwater pumps, filtration time is constrained by bottom time and decompression limits.

A promising, low-tech alternative has been proposed by using marine sponges as natural eDNA samplers ^5^. These benthic organisms filter large volumes of water daily, possess a rapid regeneration capacity, and have been successfully applied for biodiversity assessments in diverse ecosystems, including the North Atlantic ^6^, Arctic ^7^, Southern Ocean ^8, 9^, and the Indo-Pacific ^10^. These studies have shown that different sponge species exhibit varying eDNA yields, which have been attributed to differences in metabolism, pumping rates, and associated microbial communities. For instance, sponges are categorized into low microbial abundance (LMA) and high microbial abundance (HMA) ^11^, a factor influencing eDNA recovery with LMA species showing superior recovery rates ^7, 12^. Identifying the species that maximize eDNA recovery under standardized conditions is thus essential before implementing large-scale monitoring campaigns. Such comparisons, to our knowledge, have only been conducted in controlled aquaria experiments ^12^, as eDNA recovery in natural settings is subject to additional confounding factors due to actual differences in fish occupancy and environmental parameters at the time of sampling.

In the Mediterranean Sea, despite the seminal study using opportunistic samples from two local sponge species ^5^, systematic assessments of their eDNA sampling capacity are missing. The Northwestern Mediterranean façade is particularly rich in sponges, with steep walls and coralligenous habitats providing optimal conditions for sponge growth ^13^. This region also hosts several marine protected areas (MPAs) ^14^, where ongoing biomonitoring efforts could greatly benefit from novel eDNA-based approaches for biodiversity assessment. Here, we evaluated seven cosmopolitan Mediterranean sponge species thriving at a single site (coralligenous wall) to compare their effectiveness for eDNA recovery. By sampling 19 specimens under uniform environmental conditions and using a standardized processing protocol, we identified marked differences in eDNA recovery across species. Our results highlight *Axinella verrucosa* as the most promising candidate for future eDNA-based biodiversity monitoring efforts. Conversely, our findings also suggest certain species may be less effective as natural samplers, underscoring the importance of species selection in sponge-based eDNA studies.

## Methods

### Field work

**Figure 1.**
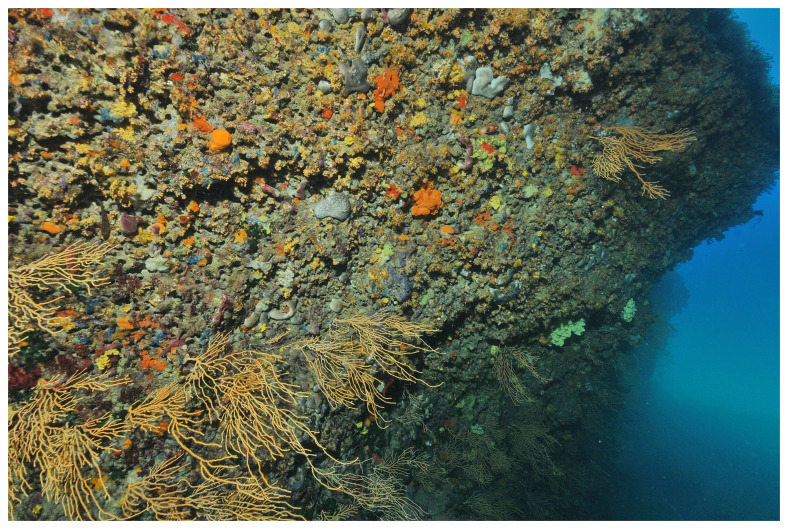
Sampling site. Underwater photography of the coralligenous wall where sponge specimens were sampled.

Sampling was conducted on May 14, 2024, at the southern tip of Frioul Island, Marseille (Cap Caveau; 43° 15.614'N / 5° 17.360'E) between 10 and 11 a.m. Three specimens were sampled for each sponge species, except for *Aplysina cavernicola* and *Axinella verrucosa*, for which only two individuals were found at the site. Biopsies were excised using a blunt-ended diving knife at a depth of 10 meters, in an area characterized by a rocky vertical wall colonized by benthic organisms, descending to a sandy plain at 14 meters ( Figure 1). Water temperature at the time of sampling was 17°C. Samples of the same species were stored in Ziploc bags within a diving mesh-bag and were transferred upon surfacing to a cooler with seawater for transport. Within 1–1.5 hours, excess water was removed, and biopsies were dried on paper tissue before being transferred into 50 mL falcon tubes with absolute ethanol. Samples were stored at -20°C until further processing.

### Sample processing

Sponge tissues (0.5 cm ^3^) were blotted dry with paper tissue, as outlined in Harper *et al.* (2023) ^15^, before being chopped with a sterile scalpel on a petri dish. DNA extraction followed a modified Qiagen Blood & Tissue Kit protocol ^16^. Briefly, sponge fragments were placed in 2 mL Eppendorf tubes and mixed with 720 µL Buffer ATL and 80 µL Proteinase K before incubation at 56°C overnight. An extraction control without starting material was included to detect potential contaminations. The next day, lysates (600 µL) were transferred to new tubes, combined sequentially with 600 µL Buffer AL and 600 µL 100% ethanol, and loaded onto DNeasy Mini spin columns in 2 mL collection tubes. After centrifugation at 6,000 × g for 1 min, the flowthroughs were discarded, and the process was repeated until all lysates had passed through the columns. The membranes were washed with 500 µL Buffer AW1 and centrifuged at 6,000 × g for 1 min, followed by a second wash with 500 µL Buffer AW2 and centrifugation at 20,000 × g for 3 min. Residual wash buffer was removed by an additional centrifugation at 20,000 × g for 1 min. DNA was eluted by adding 200 µL of preheated (56°C) AE buffer to the membranes, incubating for 1 min at room temperature, and centrifuging at 6,000 × g for 1 min. Following DNA extraction, qPCR was conducted using Fish16S primers ^17^ to assess fish eDNA presence and optimize amplification conditions. This primer set was chosen because it was recently shown to recover a greater diversity of fish species than other primer pairs in a Mediterranean site ^18^. The qPCR mix consisted of 10 µL of SsoAdvanced™ Universal SYBR ^®^ Green Supermix (BioRad), 2 µL of primers (5 µM each), 0.16 µL of BSA (Fisher), and 2 µL of DNA extract, with molecular-grade water making up a final volume of 20 µL. qPCRs were run on a CFX96 Connect Real-Time PCR Detection system (BioRad), the thermal cycling protocol included 10 minutes at 95°C, followed by 60 cycles of 30 seconds at 95°C, 30 seconds at 55°C, and 1 minute at 72°C. Samples were tested pure and diluted 10-fold and 100-fold. The 100-fold dilution performed best for all samples and was used for subsequent library preparation. Results indicated higher Fish16S detection per input DNA in the species *Clathrina clathrus* and *Agelas oroides*, which were therefore pinpointed as the best candidates and sequenced individually. The remaining samples were merged per species and sequenced as a pool. All samples were standardized to a concentration of 2.5 ng/µL to ensure uniform input for amplification. Eight technical replicates per sample were amplified using Fish16S primers tagged with unique 8-base identifiers, to allow sample dereplication during data processing. PCRs were run on a CFX96 Connect Real-Time PCR Detection system (BioRad), the reaction mix consisted of 10μL of Amplitaq Gold 360 mix (Fisher), 0.16μL of BSA (Fisher), 2μL of the primer F&R mix at 5μM each, 2μL of DNA extract and molecular grade water for a final volume of 20μL. The thermal cycling protocol included 10min at 95°C, followed by 48 cycles of 30s at 95°C, 30s at 55°C, 1min at 72°C, and a final elongation step of 7min at 72°C. Samples were purified with the MinElute kit (Qiagen), and products were controlled on a 2% agarose gel (E-Gel Power Snap ^®^, Invitrogen). The library was then constructed and sequenced by FASTERIS (Geneva, Switzerland). It was prepared with the Metafast protocol and sequencing was performed on an Illumina MiSeq platform (2 × 150 bp).

### Bioinformatic analyses

The OBITools suite ^19^ and SumaClust ^20^ were used for sequence processing and taxonomic assignment. Paired-end reads were merged (illuminapairedend), quality-filtered (obigrep), and dereplicated (obiuniq). Sequences were clustered at 97% similarity (SumaClust), with the most abundant sequence in each cluster retained as the representative cluster center. Clusters containing at least 10 reads in at least one replicate were selected for downstream analysis. Taxonomic assignment was performed with ecotag against a reference database constructed from GenBank entries using *in silico* PCR (ecoPCR) and filtered to retain sequences classified at the family level or higher (obigrep). Further filtering steps using the R package metabaR ^21^ removed chimeras (best identity <95%), contaminants (contaslayer), low-abundance artefacts (<3% relative frequency, tagjumpslayer), and replicates with sequencing coverage below 100 reads. The remaining PCR replicates were aggregated by sample, and MOTUs observed fewer than 10 times per sample were excluded. The MOTU list was then manually cured and verified with BLASTn, and MOTUs Fish16S_00009 and Fish16S_00517 assigned only to genus level were renamed as *Diplodus sp1* and *Diplodus sp2*, respectively.

## Discussion

**Figure 2.**
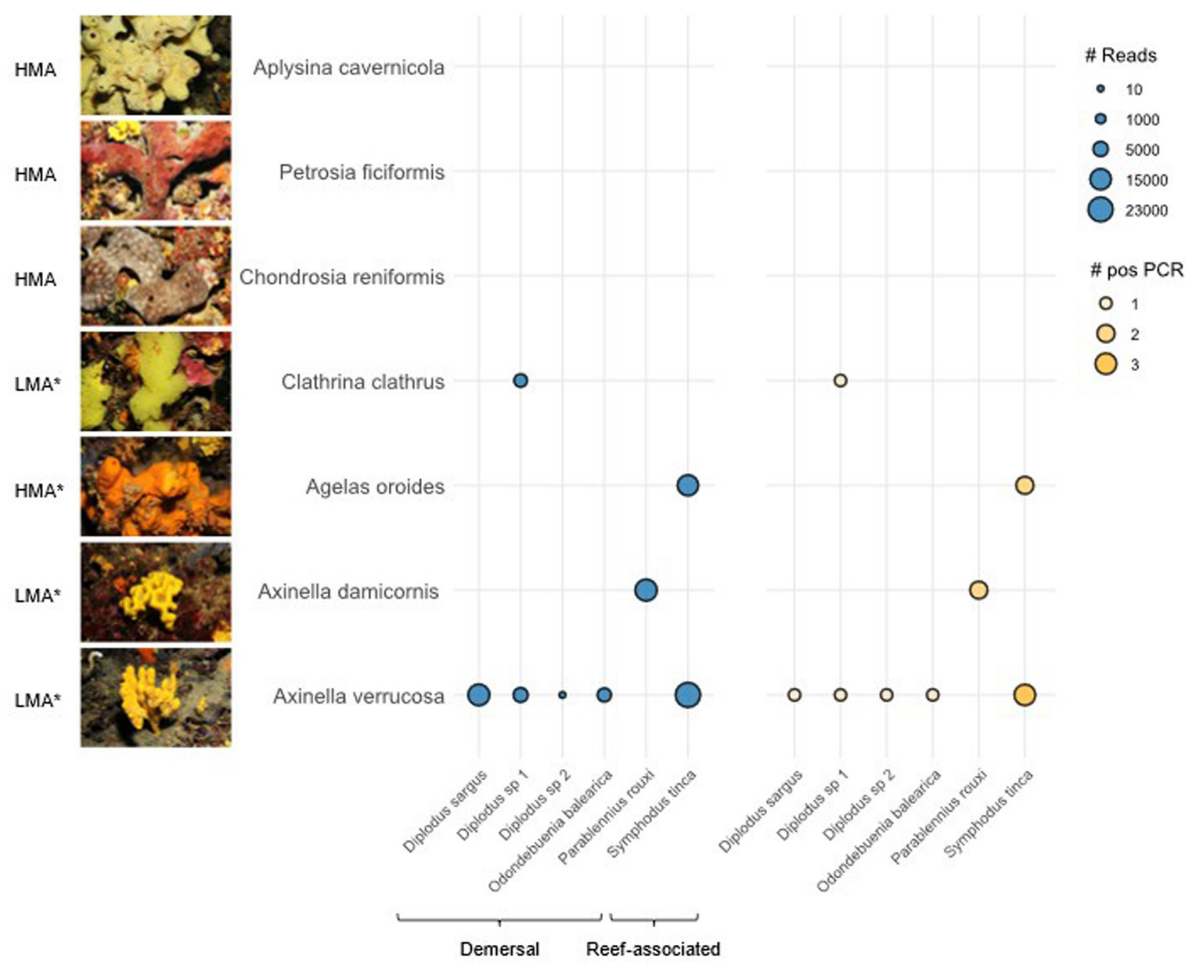
Metabarcoding results. Bubble plot depicting the number of Fish16S reads in blue (#Reads) and positive PCR replicates in orange (#pos PCR). Tested sponge species are depicted on the Y-axis with a photography of a representative specimen and their HMA/LMA status (retrieved from refs 11, 27. The * mark indicates a predicted status). Detected fish species are indicated on the X-axis with their corresponding habitat (retrieved from FishBase).

We observed marked differences in eDNA recovery across sponge species, with *Axinella verrucosa* outperforming the rest by recovering five of the six detected fish species, thus emerging as the most promising candidate for biomonitoring applications ( Figure 2). Notably, it was the only tested species with a standing 3D structure protruding into the water column, a feature that may enhance eDNA capture. With previous studies arguing both in favor ^7^ and against ^10^ a correlation between sponge morphology and eDNA recovery, this aspect warrants further investigation. The *World Porifera Database* lists 100 accepted *Axinella* species, it will be valuable to determine whether others in this genus share similar eDNA retention capabilities. A compelling candidate is *A. polypoides*, an LMA species with a standing 3D structure similar to *A. verrucosa*., though its protected status necessitates careful handling. Our findings also align with previous work showing that LMA sponges recover more eDNA than HMA sponges ^7, 12^, reinforcing the need to focus on LMA species in future studies. Including other LMA species such as *Crambe crambe* – not included in this study but known for its antimicrobial compounds ^22, 23^ and possessing a flat body - will help disentangle the influence of microbial abundance and morphology on eDNA uptake.

The detected fish community was composed of demersal and reef-associated species, highlighting the relevance of sponges as tools for benthic biodiversity monitoring. This is all the most relevant given recent findings suggesting that water eDNA is a poor proxy for benthic biodiversity ^24^, emphasizing the potential of sponges for capturing eDNA from cryptic and substrate-associated taxa. Such ability makes them particularly valuable for monitoring habitats that are difficult to access through conventional methods, such as underwater caves, crevices, and vertical walls. Moreover, while we primarily detected bottom-dwelling species, pelagic fish may be recovered upon further methodological improvements. This is supported by Turon *et al.* (2020) ^10^ who found no significant detection bias between pelagic and demersal fish when using sponge eDNA.

The overall number of detected fish species was low, and we identify several factors that may have contributed to this outcome. The sampling site lacked structural complexity and no visible fish schools were present during collection, potentially limiting the availability of eDNA in the water column. Additionally, environmental conditions at the time of sampling -such as water temperature- may have influenced DNA persistence and detection. Future campaigns targeting multiple sites and seasons, including high biodiversity MPA’s core areas, should overcome this issue. We also identify several methodological improvements that could enhance future studies. These include the immediate transfer of sponges to ethanol upon resurfacing to limit eDNA degradation, the use of multiple genetic markers to overcame reference database incompleteness and increase species detection probability ^18, 25^, and improved inhibitor removal during DNA extraction to limit sample inhibition ^26^. Finally, a key priority is the completion of reference genetic databases. Expanding the current dataset is crucial for accurate species-level assignments, particularly for commercially important taxa such as *Diplodus*, given its importance for fisheries management and conservation.

Overall, *A. verrucosa* stands out as a promising candidate for future eDNA sampling campaigns, yet protocol optimization and further testing of other LMA sponges is warranted. As eDNA-based monitoring advances, sponge sampling offers a valuable complement to water eDNA surveys, especially for benthic communities and habitats where conventional sampling is challenging. Our results arise from a preliminary pilot study for a larger monitoring scheme, yet we share them promptly to benefit MPA practitioners and inform emerging biodiversity monitoring initiatives amid growing national and international demand.

## Data Availability

Zenodo, Evaluation of Mediterranean sponges as natural samplers for environmental DNA (eDNA) - Underlying DATA. https://doi.org/10.5281/zenodo.14893662 ^28^. The project contains the following underlying data: - [Evaluation of Mediterranean sponges as natural samplers for environmental DNA (eDNA) - Underlying DATA.xls’] (comprehensive information on sample treatment and analysis steps, from DNA extraction to metabarcoding analysis). Data are available under the terms of the Creative Commons Attribution 4.0 International license (CC-BY 4.0).
