# Evaluation of Mediterranean sponges as natural samplers for environmental DNA (eDNA)

**DOI:** 10.12688/openreseurope.19755.3

**Published:** 2026-03-10

**Authors:** Nicolas Garcia-Seyda, Marielle Garcia, Dorian Guillemain, Aurélie Bonin

**Affiliations:** 1 Aix Marseille Univ, Université de Toulon, CNRS, IRD, MIO, Marseille, 13288, France; 2 Tropical Marine Ecology of Pacific and Indian Oceans - UMR 9220, Research Institute for Development (RID), Noumea, 98848, New Caledonia; 3 NGS consultancy, Marseille, 13008, France; 4 Argaly, Sainte-Hélène-du Lac, 73800, France

**Keywords:** Environmental DNA (eDNA), natural sampler DNA (nsDNA), Fish Metabarcoding, Marine sponges, Benthic biodiversity, Axinella verrucosa, Sponge-based biomonitoring

## Abstract

Marine sponges have emerged as natural samplers of environmental DNA (eDNA), offering an alternative tool for biodiversity monitoring. By filtering large volumes of seawater, they accumulate eDNA from surrounding communities and may enhance species detection where conventional water sampling is limited. Here, we evaluated seven Mediterranean sponge species co-occurring at a single site and observed differences in eDNA recovery. The number of detected fish species was consistent with other Mediterranean studies, but lower than those reported at higher latitudes. These findings highlight the need for further research and protocol optimization to fully harness Mediterranean sponges as natural eDNA samplers.

## Introduction

Environmental DNA (eDNA) has emerged as a revolutionary tool for marine biodiversity monitoring, offering a valuable complement to traditional methods based on visual surveys or species capture. ^1^ eDNA-based approaches often demonstrate superior sensitivity, particularly for detecting species that are rare, cryptic, small, nocturnal, or otherwise elusive, which are frequently missed by conventional techniques. ^2^ A significant bottleneck in current methodologies is the time-consuming water filtration process, typically restricted to surface waters due to the logistical challenges of sampling at depth. This limitation may result in the loss of valuable information on benthic biodiversity. While technological advances, such as submersible pumps and robotic sampling devices, ^3, 4^ are being developed to enable deeper sampling, they often remain prohibitively expensive. Additionally, for divers using manual underwater pumps, filtration time is constrained by bottom time and decompression limits.

A promising, low-tech alternative has been proposed by using marine sponges as natural eDNA samplers. ^5^ These benthic organisms filter large volumes of water daily, possess a rapid regeneration capacity, and have been successfully applied for biodiversity assessments in diverse ecosystems, including the North Atlantic, ^6^ Arctic, ^7^ Southern Ocean, ^8, 9^ and the Indo-Pacific. ^10^ These studies have shown that different sponge species exhibit varying eDNA yields, which have been attributed to differences in metabolism, pumping rates, and associated microbial communities. For instance, sponges are categorized into low microbial abundance (LMA) and high microbial abundance (HMA), ^11^ a factor influencing eDNA recovery with LMA species showing superior recovery rates. ^7, 12^ Identifying the species that maximize eDNA recovery under standardized conditions is thus essential before implementing large-scale monitoring campaigns. Such comparisons, to our knowledge, have only been conducted in controlled aquaria experiments, ^12^ as eDNA recovery in natural settings is subject to additional confounding factors due to actual differences in fish occupancy and environmental parameters at the time of sampling.

In the Mediterranean Sea, despite the seminal study using opportunistic samples from two local sponge species, ^5^ systematic assessments of their eDNA sampling capacity are scarce. The Northwestern Mediterranean façade is particularly rich in sponges, with steep walls and coralligenous habitats providing optimal conditions for sponge growth. ^13^ This region also hosts many marine protected areas (MPAs), ^14^ where ongoing biomonitoring efforts could greatly benefit from novel eDNA-based approaches for biodiversity assessment. Here, we evaluated seven cosmopolitan Mediterranean sponge species thriving at a single site (coralligenous wall) to compare their effectiveness for eDNA recovery. By sampling 19 specimens collected in close proximity under uniform environmental conditions, and by using a standardized processing protocol, we observed differences in eDNA recovery across species. The number of detected fish species was consistent with other reports from the Mediterranean Sea, yet lower than those reported for sponge-based studies at higher latitudes. These findings highlight the need for further research and protocol optimization to fully harness Mediterranean sponges as natural eDNA samplers.

## Methods

### Field work

**Figure 1.**
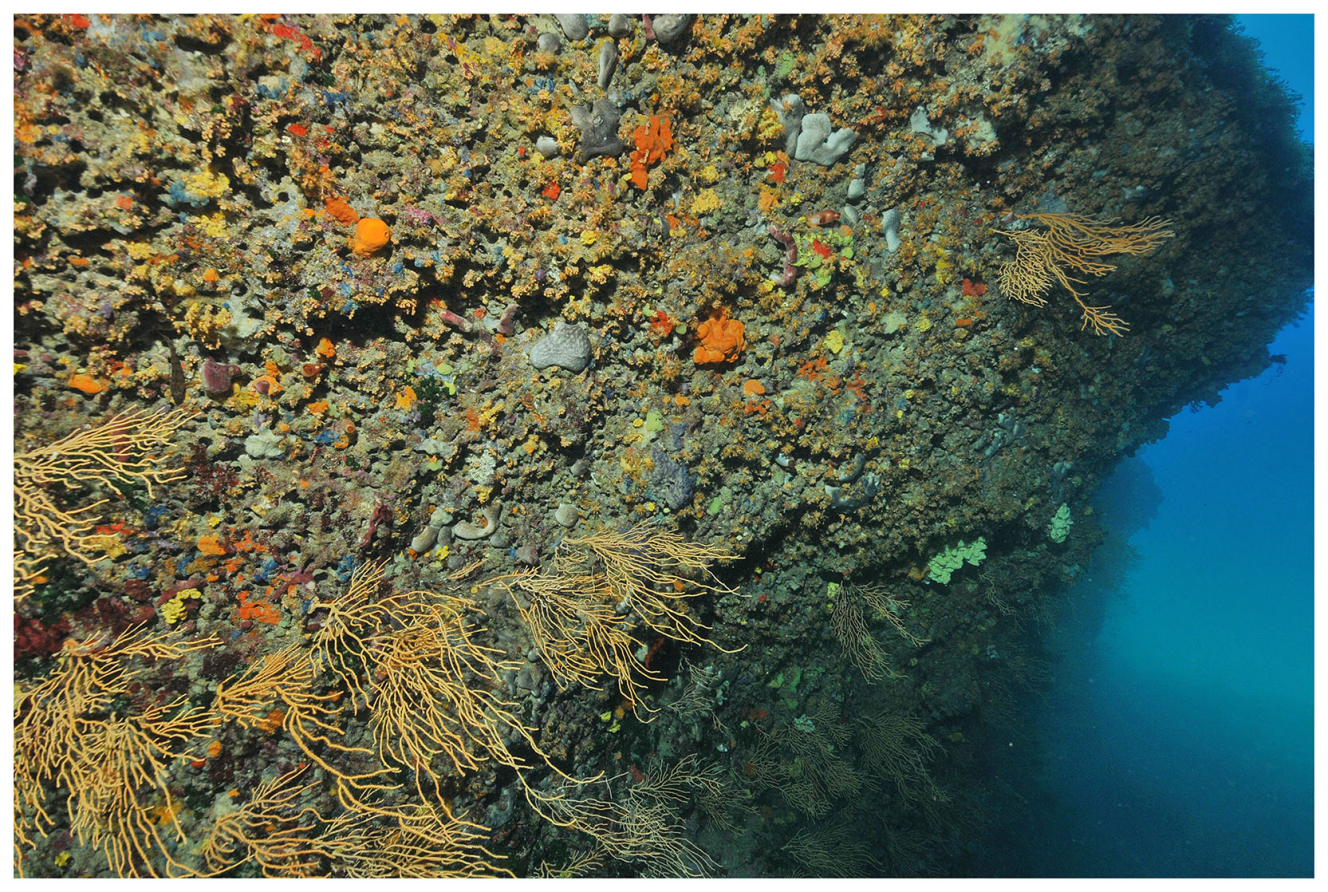
Sampling site. Underwater photography of the coralligenous wall where sponge specimens were sampled.

Sampling took place on May 14, 2024, between 10 and 11 a.m. at Cap Caveau, Frioul Island, Marseille (43°15.614’N/5°17.360′E). The list of sampled species was: *Clathrina clathrus*, *Chondrosia reniformis*, *Petrosia ficiformis*, *Agelas oroides*, *Axinella damicornis*, *Aplysina cavernicola*, and *Axinella verrucosa.* Biopsies were excised using a blunt-ended diving knife at a depth of 10 meters on a rocky vertical wall colonized by benthic organisms and descending to a sandy plain at 14 m ( Figure 1). All sponges were collected within a 10 m distance to minimize location bias. Three individuals were sampled per species, except for *A. cavernicola* and *A. verrucosa*, for which only two were found within the sampling distance. The campaign was designed as an initial screening to identify candidate sponge species for further optimization; therefore, to minimize logistical and analytical costs, biological replicates were pooled into a single sample per species. Water temperature at the time of sampling was 17 °C. Specimens of the same species were stored in Ziploc bags inside a mesh bag during collection, and transferred upon surfacing to a seawater-filled cooler. Within 1–1.5 h, excess water was removed, biopsies were blotted dry, and samples were preserved in 50 mL Falcon tubes with absolute ethanol before storage at −20 °C until further processing.

### Sample processing

Sponge tissues (0.5 cm ^3^) were blotted dry with paper tissue, as outlined in Harper *et al.* (2023), ^15^ before being chopped with a sterile scalpel on a petri dish. DNA extraction followed a modified Qiagen Blood & Tissue Kit protocol. ^16^ Briefly, sponge fragments were placed in 2 mL Eppendorf tubes and mixed with 720 μL Buffer ATL and 80 μL Proteinase K before incubation at 56 °C overnight. An extraction control without starting material was included to detect potential contaminations. The next day, lysates (600 μL) were transferred to new tubes, combined sequentially with 600 μL Buffer AL and 600 μL 100% ethanol, and loaded onto DNeasy Mini spin columns in 2 mL collection tubes. After centrifugation at 6,000 × g for 1 min, the flowthroughs were discarded, and the process was repeated until all lysates had passed through the columns. The membranes were washed with 500 μL Buffer AW1 and centrifuged at 6,000 × g for 1 min, followed by a second wash with 500 μL Buffer AW2 and centrifugation at 20,000 × g for 3 min. Residual wash buffer was removed by an additional centrifugation at 20,000 × g for 1 min. DNA was eluted by adding 200 μL of preheated (56 °C) AE buffer to the membranes, incubating for 1 min at room temperature, and centrifuging at 6,000 × g for 1 min.

Following DNA extraction, qPCR was conducted using Fish16S primers ^17^ to assess fish eDNA presence and optimize amplification conditions. This primer set was chosen because it was recently shown to recover a greater diversity of fish species than other primer pairs in a Mediterranean site. ^18^ The qPCR mix consisted of 10 μL of SsoAdvanced™ Universal SYBR ® Green Supermix (BioRad), 2 μL of primers (5 μM each, Sigma), 0.16 μL of BSA (Fisher), and 2 μL of DNA extract, with molecular-grade water making up a final volume of 20 μL. qPCRs were run on a CFX96 Connect Real-Time PCR Detection system (BioRad), the thermal cycling protocol included 10 minutes at 95 °C, followed by 60 cycles of 30 seconds at 95 °C, 30 seconds at 55 °C, and 1 minute at 72 °C. Samples were tested pure and diluted 10-fold and 100-fold. The 100-fold dilution performed best for all samples and was used for subsequent library preparation. Samples were normalized to 2.5 ng/μL to ensure uniform input for amplification and, given the screening design of the study, pooled by species for sequencing. The only exceptions were *C. clathrus* and *A. oroides*, which exhibited the lowest Cq values (potentially indicating stronger Fish16S detection) and were thus sequenced individually to generate rarefaction curves.

Eight technical replicates per sample were amplified using Fish16S primers tagged with unique 8-base identifiers, to allow sample dereplication during data processing. PCRs were run on a CFX96 Connect Real-Time PCR Detection system (BioRad), the reaction mix consisted of 10 μL of Amplitaq Gold 360 mix (Fisher), 0.16 μL of BSA (Fisher), 2 μL of the primer F&R mix at 5 μM each (Sigma), 2 μL of DNA extract and molecular grade water for a final volume of 20 μL. The thermal cycling protocol included 10 min at 95 °C, followed by 48 cycles of 30s at 95 °C, 30s at 55 °C, 1 min at 72 °C, and a final elongation step of 7 min at 72 °C. Samples were purified with the MinElute kit (Qiagen), and products were verified on a 2% agarose gel (E-Gel Power Snap ®, Invitrogen). The library was then constructed and sequenced by FASTERIS (Geneva, Switzerland). It was prepared with the Metafast protocol and sequencing was performed on an Illumina MiSeq platform (2 × 150 bp).

PCR controls were added to the experiment to monitor potential contaminations and to verify the success of the amplification and sequencing. Negative PCR controls (samples without DNA) were named “CPCR001” and “CPCR002”. Positive PCR controls included a synthetic oligonucleotide (“CPOS124”, leading to a low best identity value because it corresponds to a random DNA sequence) and a freshwater environmental DNA extract (“CPOS125”) which showed positive results in previous experiments with the Fish16S marker. Additionally, bioinformatic controls (“BLNK”, combinations of primer tags that were not present in the experiment but were followed bioinformatically) were used to monitor the level of tag jumps. ^18*^

### Bioinformatic analyses

The OBITools suite (version 2) ^19^ and SumaClust ^20^ were used for sequence processing and taxonomic assignment. Paired-end reads were merged (illuminapairedend), quality-filtered (obigrep), and dereplicated (obiuniq). Sequences were clustered at 97% similarity (SumaClust), with the most abundant sequence in each cluster retained as the representative cluster center. Clusters containing at least 10 reads in at least one replicate were selected for downstream analysis. Taxonomic assignment was performed with ecotag against a reference database constructed from GenBank (release 249) entries using *in silico* PCR (ecoPCR) and filtered to retain sequences classified at the family level or higher (obigrep). Further filtering steps using the R package metabaR ^21^ removed chimeras (best identity <95%), contaminants (contaslayer), low-abundance artefacts (<3% relative frequency, tagjumpslayer), and replicates with sequencing coverage below 100 reads. The remaining PCR replicates were aggregated by sample, and MOTUs observed fewer than 10 times per sample were excluded. Finally, the MOTU list was manually cured and verified with BLASTn, and MOTUs Fish16S_00009 and Fish16S_00517 assigned only to genus level were renamed as *Diplodus sp1* and *Diplodus sp2*, respectively. *A. cavernicola*, *Petrosia ficiformis*, and *C. reniformis* did not yield any sequences after bioinformatic processing. For the species sequenced individually, *C. clathrus* and *A. oroides*, sequences were recovered from only one specimen each, preventing the generation of rarefaction curves.

## Discussion

**Figure 2.**
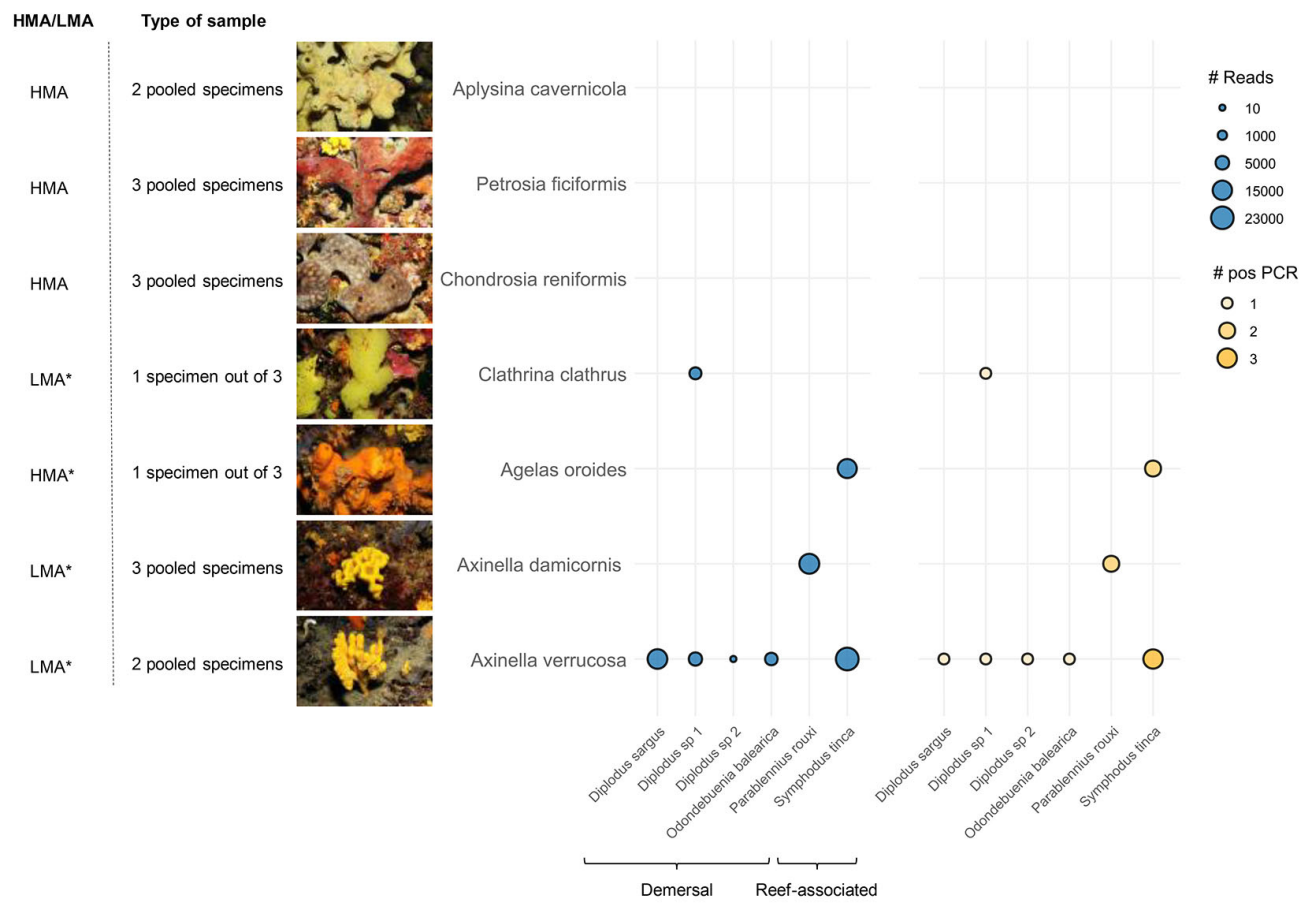
Metabarcoding results. Bubble plot depicting the number of Fish16S reads in blue (#Reads) and positive PCR replicates (#pos PCR, out of 8 replicates) in orange. Tested sponge species are depicted on the Y-axis with an image of a representative specimen, their HMA/LMA status (retrieved from refs 11, 25. The * mark indicates a predicted status) and the type of sample analyzed. Detected fish species are indicated on the X-axis with their corresponding habitat (retrieved from FishBase). *A. cavernicola*, *Petrosia ficiformis*, and *C. reniformis* did not yield any sequences after bioinformatic processing. For *C. clathrus* and *A. oroides*, sequences were obtained from only one of the three specimens sequenced.

We observed differences in eDNA recovery across sponge species, as *Axinella verrucosa* recovered five taxa, three assigned at the species and two at the genus level, while *A. cavernicola*, *P. ficiformis,* and *C. reniformis* did not recover any sequence ( Figure 2). Notably, *A. verrucosa* was the only species tested with a standing 3D structure protruding into the water column, a feature that may enhance eDNA capture. With previous studies arguing both in favor ^7^ and against ^10^ a correlation between sponge morphology and eDNA recovery, this aspect warrants further investigation. The *World Porifera Database* lists 100 accepted *Axinella* species, it will be valuable to determine whether others in this genus share similar or better eDNA retention capabilities. Our findings seem to align with previous work showing that LMA sponges recover more eDNA than HMA sponges, ^7, 12^ underscoring the need to focus on LMA species in future studies. Including other LMA species such as *Crambe crambe* – not included in this study but known for its antimicrobial compounds ^22, 23^ and possessing a flat body - may help disentangle the influence of microbial abundance and morphology on eDNA uptake.

The fish detected belonged to demersal and reef-associated species, suggesting that sponges may offer potential as tools for monitoring the seabed. This is particularly relevant in light of recent findings indicating that water eDNA can be a poor proxy for benthic biodiversity, ^24^ emphasizing the potential of sponges for capturing eDNA from substrate-associated taxa. Such ability could make them particularly valuable for monitoring habitats that are difficult to access through conventional methods, such as underwater caves, crevices, and vertical walls. Moreover, while we only detected bottom-dwelling species, pelagic fish might be recovered upon further methodological improvements. This is supported by Turon *et al.* (2020) ^10^ who found no significant detection bias between pelagic and demersal fish when using sponge eDNA.

The overall number of detected fish species was low, yet consistent with previous reports for Mediterranean sponges. Mariani (2019) ^5^ detected four and seven fish taxa from *Ircinia fasciculata* and *P. ficiformis* specimens, respectively, sampled at two different sites and analyzed with 12S primers (Teleo02). Corral-Lou (2025) ^26^ reported six fish taxa from 32 specimens of *Haliclona cinerea* and *Haliclona mediterranea*, sampled across four sites in two different seasons, and analyzed with COI primers (mlCOIintF-XT/jgHCO2198). More recently, Neave et al. (in press) ^27^ reported 13 fish taxa from 85 specimens belonging to four sponge species - *Chondrosia reniformis, Sarcotragus* sp., *Ircinia variabilis* and *Ircinia* sp. - analyzed with Teleo02 primers. Such consistently low detections raise the question of whether environmental conditions exert a stronger influence on Mediterranean sponges than on those from higher latitudes. One possible explanation is the relatively shallow depth at which Mediterranean sponge studies are typically conducted, which may accelerate eDNA degradation due to increased irradiance ^28^ and warmer seawater. ^29^

In our study, Fish16S primers appeared to generate artifactual amplifications, as indicated by the numerous MOTUs with low best identity values (<0.65 in Table 6 in the Underlying Data), particularly in negative controls and low-input sponge samples. This likely explains why *Chondrosia clathrus* and *Aplysina oroides* samples showed high apparent fish eDNA ratios (Table 3 in the Underlying Data) but few validated reads after filtering, highlighting the critical influence of primer choice on fish detection in sponge-derived eDNA. Additional ecological factors may have further limited detections. The sampling site lacked structural complexity, and no fish schools were observed during collection, likely limiting eDNA availability in the surrounding water column. Temporal environmental conditions at the time of sampling may also have affected eDNA persistence and detectability. Future surveys targeting multiple locations and seasons, including biodiversity-rich marine protected areas (MPAs), may help disentangle these effects.

Several methodological refinements could improve detection rates in the future. These include the immediate preservation of sponges in ethanol upon resurfacing to limit eDNA degradation, the use of multiple genetic markers - particularly the 12S marker - to mitigate reference database limitations and improve taxonomic resolution, ^18, 30^ and enhanced inhibitor removal during DNA extraction to reduce PCR inhibition. ^31^ Finally, expanding current reference databases remains a key priority. This is especially critical for ecologically and economically important groups such as *Diplodus*, where accurate species-level identification is essential for effective fisheries management and conservation planning.

As eDNA-based monitoring advances, sponge sampling may provide a valuable complement to water-based surveys, particularly for benthic habitats where conventional sampling is challenging. However, protocol refinement, broader testing across additional LMA sponges, and a better understanding of the drivers of low detections in Mediterranean sponges, remain necessary. Although our findings stem from a preliminary pilot study for a larger monitoring scheme, we share them promptly to support MPA practitioners and inform emerging biodiversity monitoring initiatives amid growing national and international demand.

### Limitations of the study

Because of its screening nature and the lack of biological replicates, the study design limits statistical analysis and prevents a reliable determination of whether *A. verrucosa* or LMA sponges are better candidates for eDNA sampling than their HMA counterparts. Nevertheless, it shall guide future monitoring initiatives by indicating which species are less suitable for testing and by signaling those that warrant further optimization and refinement.

## Data Availability

Zenodo, Evaluation of Mediterranean sponges as natural samplers for environmental DNA (eDNA) - Underlying DATA V2. https://doi.org/10.5281/zenodo.17184712. ^32^ The project contains the following underlying data: - [Evaluation of Mediterranean sponges as natural samplers for environmental DNA (eDNA) - Underlying DATA.xls’] (comprehensive information on sample treatment and analysis steps, from DNA extraction to metabarcoding analysis). - [ Sequencing raw data.zip] (zip file containing the raw sequencing data, arising from a shared library, with an ngs filter with this project’s tags to reassign sequences to samples). Data are available under the terms of the Creative Commons Attribution 4.0 International license (CC-BY 4.0).
