# Supplementary material for: Evaluation of Mediterranean sponges as natural samplers for environmental DNA (eDNA)
Source: Open Res Eur. 2026 Mar 10;5:69. Originally published 2025 Mar 17. [Version 3] doi: 10.12688/openreseurope.19755.3 (PMC13176835; doi:10.12688/openreseurope.19755.3)
Supplement: Peer review history

# Reviewer report: Reviewer response for version 3

**DOI:** 10.21956/openreseurope.25200.r73647

**Published:** 2026-05-14

**Authors:** Soo-Je Park

**Affiliations:** 1 Jeju National University, Jeju-si, South Korea

The (revised) manuscript showed an interesting and timely pilot evaluation of Mediterranean sponge species as natural samplers for eDNA-based biodiversity monitoring. This study provides useful methodological observations and practical considerations for future sponge-based biomonitoring efforts in various marine environments. In particular, the manuscript is well organized, the experimental workflow is clearly described, and the raw sequencing data are openly available, which greatly enhances reproducibility. Overall, this work is appropriate as a brief report and serves as a valuable data description–style resource for future optimization studies in marine eDNA monitoring.

 Minor comments:

 Figure 2 is informative and visually well designed. However, the readability could be improved by slightly enlarging the fish species labels on the X-axis and clarifying the distinction between “#Reads” and “#pos PCR” in the legend.

Is the study design appropriate and does the work have academic merit?

Yes

Is the work clearly and accurately presented and does it cite the current literature?

Yes

If applicable, is the statistical analysis and its interpretation appropriate?

Partly

Are all the source data underlying the results available to ensure full reproducibility?

Yes

Are the conclusions drawn adequately supported by the results?

Yes

Are sufficient details of methods and analysis provided to allow replication by others?

Yes

Reviewer Expertise:

ecogenomics, metagenomics, environmental microbiology, microbial ecology

I confirm that I have read this submission and believe that I have an appropriate level of expertise to confirm that it is of an acceptable scientific standard.

# Reviewer report: Reviewer response for version 3

**DOI:** 10.21956/openreseurope.25200.r71035

**Published:** 2026-04-30

**Authors:** Rachael Ununuma Chidugu-Ogborigbo

**Affiliations:** 1 University of the West of England, Bristol, UK

**Reviewer report**

 Garcia-Seyda et al. report on a comparison of seven Mediterranean sponge species as natural eDNA samplers for fish detection, using Fish16S metabarcoding on specimens taken from a single coralligenous wall site near Marseille. The question they are asking is a useful one: do sponges differ in how much fish eDNA they capture, and does microbial load (LMA versus HMA status) help explain those differences? There is real practical value in getting this right, particularly for MPA monitoring in the Mediterranean, where conventional visual surveys are costly and logistically difficult. I want to be clear that I think the topic deserves to be pursued. My concerns are about whether this dataset is ready to support the conclusions the authors draw from it.

 I have read the manuscript at version 3 alongside the full revision history and the prior reports from Ruiz-Ramos, Gallego, Hoban, and Dowell. The authors have responded to reviewers in good faith, softened some of the stronger claims, added a Limitations section, and made their raw data available. I recognise all of that. The difficulty is that the issues raised since version 1 go deeper than tone, and revising the language around a finding does not change what the data can and cannot show.

**Study design and evidential basis**

 The core problem is straightforward: because biological replicates were pooled before sequencing, there is effectively one sequenced library per sponge species. For *A. verrucosa*, the species that produced the only substantive sequencing result, only two individuals were collected in the first place. The main claim of the paper rests on a comparison between a single pooled library from two *A. verrucosa* specimens and pooled libraries from the other species. There is no way to test statistically whether that difference is real, or a product of the individuals sampled, the pooling process, or chance. The authors are right that pooling is standard practice in early-stage screening, and I do not object to that decision per se; what I object to is treating the output of a screening exercise as evidence for a species-level conclusion.

 There is also a sampling asymmetry worth noting. Five species were represented by three individuals each, while *A. cavernicola* and *A. verrucosa* had only two. The species with the smallest sample produced the headline result. That is not necessarily a problem, but it is not discussed anywhere in the manuscript, and it adds another layer of uncertainty to any comparison between species.

**Primer performance and internal inconsistency**

 The Fish16S primers caused real problems here. A large proportion of MOTUs had best-identity values below 0.65, particularly in the negative controls and in samples with low template input. More tellingly, the two species with the strongest qPCR signal (C. clathrus and A. oroides) returned almost nothing after bioinformatic filtering. When qPCR and sequencing results go in opposite directions like this, something has gone wrong, and the most likely explanation the authors now offer is that the primers were generating artefactual amplifications. That explanation is plausible, but it raises an obvious question: why was no alternative primer set run in parallel? The Teleo02 12S primers have been used successfully in several of the Mediterranean studies the authors cite, and running a second marker would have provided a cross-check. Using a single primer set that turns out to behave erratically in this sample matrix is a design weakness that cannot be addressed retrospectively in the Discussion.

 This matters especially for the three species that returned no sequences at all ( *A. cavernicola, P. ficiformis*, *C. reniformis*). The authors state that all samples were sequenced, but some returned nothing after bioinformatic cleaning. That may be true, but given the primer behaviour described above, it is genuinely unclear whether those zeros reflect poor eDNA capture by those species, PCR inhibition, filtering artefacts, or some combination. The paper presents them as biological negatives without adequately considering the alternatives.

Taxonomic resolution

 Five taxa were recovered from *A. verrucosa*, but two of them could only be assigned to genus level as Diplodus sp.1 and sp.2. The authors explain clearly why species-level assignment was not possible given the similarity within that genus, which I appreciate. But it does mean the best-case tally from the top-performing species in this study is three confirmed species identifications. The paper makes much of the potential value of sponge eDNA for monitoring commercially important fish like Diplodus; that argument depends on being able to tell species apart, which the current primer and reference database combination cannot reliably do.

The HMA/LMA interpretation

 The HMA/LMA framework is used as the main explanatory lens for the results, and the Discussion suggests the findings are consistent with LMA sponges being better eDNA collectors. The difficulty is that the HMA/LMA designations for all seven species are predicted, not measured, as the asterisks in Figure 2 make clear. They come from machine learning classifications in the cited literature, not from direct characterisation of the specimens used here. It is not straightforward to argue that results support a hypothesis about microbial abundance when the microbial abundance itself was not measured. If the authors want to make this argument in a future, better-powered study, some direct assessment of microbial load in the tested specimens would be necessary.

The introduction

 The introduction reads as a general primer on eDNA metabarcoding and sponge sampling rather than a focused rationale for this study. I would find it more useful to know why these seven species were chosen, what is known about their pumping rates or tissue architecture, and what predictions one might reasonably make about their eDNA capture performance before seeing the results. Without that, it is hard to judge whether the observed ranking makes biological sense or is simply a product of the specific conditions on the day of sampling.

What works

 The field rationale is sound, and the gap being addressed is real: there are very few systematic in situ comparisons of Mediterranean sponge species for eDNA recovery. The coralligenous habitat is ecologically important and undermonitored. The laboratory methods are clearly described and reproducible. The bioinformatic pipeline is standard and appropriate. Making the raw sequencing data available on Zenodo is the right thing to do, and I am glad it was done. Contextualising the detection numbers against prior Mediterranean studies is a fair approach and helps put the low counts in perspective. The decision to publish preliminary findings openly and promptly, rather than waiting for a larger and more complete study, is defensible and potentially useful for practitioners.

Summary

 My honest assessment is that this dataset is not yet sufficient to support publication, even as a brief report. The pooled design means the headline finding cannot be tested. The primer artefacts mean the negative results are uninterpretable. The HMA/LMA interpretation rests on predicted rather than measured microbial status. None of this can be fixed by further revision of the text. What is needed is the follow-up study the authors say is planned: independent replicates, at least two primer sets run in parallel, and some empirical characterisation of the microbial communities in the tested sponges. That study would be publishable. This one, in my view, is not yet.

Is the study design appropriate and does the work have academic merit?

Partly

Is the work clearly and accurately presented and does it cite the current literature?

Partly

If applicable, is the statistical analysis and its interpretation appropriate?

Not applicable

Are all the source data underlying the results available to ensure full reproducibility?

Yes

Are the conclusions drawn adequately supported by the results?

No

Are sufficient details of methods and analysis provided to allow replication by others?

Yes

Reviewer Expertise:

Marine biodiversity, environmental Science, benthic ecology, sponge biology

I confirm that I have read this submission and believe that I have an appropriate level of expertise to state that I do not consider it to be of an acceptable scientific standard, for reasons outlined above.

# Reviewer report: Reviewer response for version 2

**DOI:** 10.21956/openreseurope.23255.r67850

**Published:** 2026-01-19

**Authors:** Rosie Dowell

**Affiliations:** 1 Liverpool John Moores University, Liverpool, UK

In this brief report, Garcia-Seyda et al. evaluate the effectiveness of seven marine sponges as natural eDNA samplers for the detection of fish in the Mediterranean Sea. 

 While I understand the authors intentions in publishing preliminary data early and appreaciate the changes that have been made to not overstate the promise of A. Verrucosa, I do not believe this report at this stage adds useful information to the field. I assume further optimisation of select sponge species in underway after these preliminary stages and will inform a more thorough manuscript. 

 I also believe the introduction is very general and would benefit from more detailed discussion of the chosen sponges and the potential applications of nsDNA in this ecosystem.

Is the study design appropriate and does the work have academic merit?

Partly

Is the work clearly and accurately presented and does it cite the current literature?

Yes

If applicable, is the statistical analysis and its interpretation appropriate?

Not applicable

Are all the source data underlying the results available to ensure full reproducibility?

Yes

Are the conclusions drawn adequately supported by the results?

No

Are sufficient details of methods and analysis provided to allow replication by others?

Yes

Reviewer Expertise:

Marine eDNA metabarcoding

I confirm that I have read this submission and believe that I have an appropriate level of expertise to state that I do not consider it to be of an acceptable scientific standard, for reasons outlined above.

# Reviewer report: Reviewer response for version 2

**DOI:** 10.21956/openreseurope.23255.r61721

**Published:** 2025-12-24

**Authors:** Dannise V. Ruiz-Ramos

**Affiliations:** 1 University of Maryland Eastern Shore, Princess Anne, MD, USA

I appreciate the efforts to improve the manuscript and the inclusion of the Limitations of the Study paragraph. I understand this is a brief report and statistics are limited. However, the introduction remains misleading, suggesting that A. verrucosa would be a good natural sampler, but the statement is unsupported. Even when A. verrucosa "appears to recover more fish species", it only recovers five species; therefore, sponges might be inefficient eDNA samplers. Still, the report's tone fails to articulate that. 

 Additionally, if results vary by sponge species, what would be the effect of sampling a particular sponge species for eDNA monitoring? This problem is somewhat acknowledged in the discussion "A compelling candidate is *A. polypoides*, an LMA species with a standing 3D structure similar to *A. verrucosa*, though its protected status necessitates careful handling." I am not familiar with the conservation status of sponges in the Mediterranean, but I would advise caution when promoting the collection of a species for biomonitoring.

Is the study design appropriate and does the work have academic merit?

Partly

Is the work clearly and accurately presented and does it cite the current literature?

Yes

If applicable, is the statistical analysis and its interpretation appropriate?

No

Are all the source data underlying the results available to ensure full reproducibility?

Partly

Are the conclusions drawn adequately supported by the results?

No

Are sufficient details of methods and analysis provided to allow replication by others?

Partly

Reviewer Expertise:

Marine biology, environmental DNA, environmental omics.

I confirm that I have read this submission and believe that I have an appropriate level of expertise to state that I do not consider it to be of an acceptable scientific standard, for reasons outlined above.

# Response

**Published:** 2026-01-06

**Authors:** Nicolas Garcia Seyda

**Affiliations:** Institute of Research for Development, Marseille, France

We thank the reviewer for the time dedicated to evaluating our revised manuscript. We reply in a point-by-point manner:  

**COMMENT 1:** the introduction remains misleading, suggesting that A. verrucosa would be a good natural sampler, but the statement is unsupported.

 RESPONSE 1: In our revised version, all claims have been downgraded following the reviewer’s initial concerns:

- *A. verrucosa* is not mentioned in the title (it was not in the first version either),
- In the abstract, we state very cautiously that (1) ‘We observed **differences in eDNA recovery** across tested species’, (2) ‘ *A. verrucosa* **appears** to recover more fish eDNA’, and (3) ‘it stands out as the best candidate **for** **further optimization’**.
- In the introduction, we carefully state ‘Our results highlight *Axinella verrucosa* as the most promising candidate which, **after further optimization**, **may become** an alternative tool for eDNA-based biodiversity monitoring’. 
- In the discussion, we once again begin by stating ‘We observed **differences in eDNA recovery **across sponge species’, and the article ends ‘Overall, *A. verrucosa* **appears** to recover more fish eDNA making it a **promising candidate **for **further protocol refinement**. However, **optimization efforts** […] **remain warranted**.

Therefore, the main claim is not that *A. verrucosa* is a good natural sampler *per se*, but rather that A. verrucosa is **a good candidate for** **future R&D**.  

**COMMENT 2: **Even when A. verrucosa "appears to recover more fish species", it only recovers five species; therefore, sponges might be inefficient eDNA samplers. Still, the report's tone fails to articulate that.

 RESPONSE 2: The reviewer’s main critic, challenging the report’s tone and claiming that the introduction is misleading, seems to arise from the general premise that sponges are inefficient eDNA samplers. However, the positive tone as noted in the introducing sentence: ‘A promising, low-tech alternative has been proposed by using marine sponges as natural eDNA samplers’, is based not on the presented results but on the cited fast-growing body of evidence in which marine sponges have been used to monitor biodiversity in the Mediterranean (Mariani 2019, Corral-Lou 2025), North Atlantic (Neave 2005), Arctic (Brodnicke  2023), Indo-Pacific (Turon 2020), and Southern Ocean (Jeunen 2023, Jeunen  2024). Moreover, as now added to the discussion, our results are consistent with previous reports for Mediterranean sponges. Mariani et al. (2019) detected 4 and 7 fish taxa from *2 *sponge specimens sampled at two different sites, and Corral-Lou et al. (2025) reported six fish taxa (with 4 identified to species level) despite a greater sampling effort consisting of 32 sponge specimens sampled across four sites and two different seasons. Therefore, our results arising from a lower sampling effort, with sponges sampled at a single site and a single season, are rather a success than a failure. All in all, the report’s positive tone is grounded in the established and growing literature on sponge-based eDNA sampling, and the reviewer’s concern appears to reflect a broader debate rather than a limitation specific to the present study.  

**COMMENT 3:** Additionally, if results vary by sponge species, what would be the effect of sampling a particular sponge species for eDNA monitoring?

 RESPONSE 3: As noted in this study and by the cited literature, notably by Cai et al. (2022), each sponge species exhibits a different eDNA yield due to differences in metabolism, pumping rates, and associated microbial communities. Therefore, it is crucial to screen and identify the sponge species that maximize eDNA recovery. Conversely, identifying species with low eDNA recovery may inform which species to avoid in the future.

**COMMENT 4:** I am not familiar with the conservation status of sponges in the Mediterranean, but I would advise caution when promoting the collection of a species for biomonitoring.

 RESPONSE 4: The reference to *A. polypoides* in the Discussion is intended to illustrate how sponge height and exposure to the water column may influence eDNA recovery, rather than promoting the targeted collection of this species for biomonitoring. *A. polypoides* individuals are typically an order of magnitude taller than *A. verrucosa* (50 cm vs 5 cm), increasing their exposure to hydrodynamic flow and potentially capturing more eDNA. In contrast, a flat species such as *Crambe crambe* may be less exposed to currents and thus capture less eDNA. Accordingly, a hypothetical study comparing eDNA recovery rates between sponges with contrasting morphologies (e.g., *A. polypoides*, *A. verrucosa*, and *C. crambe*) is proposed as a conceptual example to disentangle the role of sponge height and water-column exposure in eDNA recovery. Such understanding would inform sampling initiatives based on passive samplers, such as the metaprobe (Maiello 2022), as placing filters too close to the substrate may limit the amount of captured eDNA, yet placing it too far may limit the amount of detected benthic species. Finally, we note that sponge tissue requirements for eDNA analyses are minimal (0.5 cm³, ~50 mg), and sponges are known for their rapid healing capacity (within few days/weeks, healing dynamics are currently being studied for *A. verrucosa*). Nevertheless, we agree that the conservation status must be carefully considered, and any future application of sponge-based biomonitoring should adhere strictly to local protection regulations and ethical sampling guidelines.

# Reviewer report: Reviewer response for version 2

**DOI:** 10.21956/openreseurope.23255.r61924

**Published:** 2025-11-20

**Authors:** Mykle Hoban

**Affiliations:** 1 Hawaii Institute of Marine Biology, Kaneohe, Hawaii, USA

In this short note, the authors evaluate various species of Mediterranean sponges for their efficacy as natural eDNA samplers. They find that *Axinella verrucosa *appears to recover more taxa than other species tested. 

 I am satisfied by the authors' responses to the initial round of reviews and their associated updates to the manuscript. The study lacks complexity and would certainly benefit from greater sampling depth and statistical power, but the authors address these limitations in their revisions and as a preliminary report, I believe it has merit.

Is the study design appropriate and does the work have academic merit?

Yes

Is the work clearly and accurately presented and does it cite the current literature?

Yes

If applicable, is the statistical analysis and its interpretation appropriate?

Not applicable

Are all the source data underlying the results available to ensure full reproducibility?

Yes

Are the conclusions drawn adequately supported by the results?

Yes

Are sufficient details of methods and analysis provided to allow replication by others?

Yes

Reviewer Expertise:

Environmental DNA, fish biology & taxonomy, marine biodiversity

I confirm that I have read this submission and believe that I have an appropriate level of expertise to confirm that it is of an acceptable scientific standard.

# Reviewer report: Reviewer response for version 1

**DOI:** 10.21956/openreseurope.21370.r52560

**Published:** 2025-05-19

**Authors:** Ramon Gallego

**Affiliations:** 1 Universidad Autónoma de Madrid, Madrid, Spain

The brief report by Garcia-Seyda and collaborators touches on a timely topic in marine biodiversity surveys - are there some sponge species that are better at capturing environmental DNA than others? And is it a function of their microbiome abundance? The topic is interesting but there are a few methodological choices that make it less useful, and the conclusions dubious.

 The authors sample 19 specimens from seven species of sponges from the same environment. One first drawback is that (methods section, first paragraph) "Samples of the same species were stored in Ziploc bags". In my opinion that gets rid of the value of replicates, and now you are left with seven samples. 

 The authors do their best for keeping extraction protocols standard across samples, although one can only imagine it is hard to standardize 0.5cm3 of tissue. 

 The authors do a qPCR using fish-specific primers to estimate the amount of fish target DNA inside the sponges. Not mentioned in the main report, but the supplementary material states all samples are in a similar range of fish DNA concentration. The authors then treated the species with bigger numbers (no statistical test for differential concentration of fish DNA) differently to the rest, which they pooled in a single tube per sponge species. I think this is a bad choice. 

 The sequencing results are not very promising, with only 6 fish species detected in 4 of the 7 sponge pools. This low diversity goes against the results shown in the qPCR assay, and all previous experiments on eDNA retention in sponges, suggesting something must have gone wrong on either the library preparation, the sequencing or the analysis. 

 It is totally possible that Axinella spp. are better eDNA samplers than the other species collected here. However, the results shown are not enough to support or disregard this assumption. 

 I would encourage them to share the raw sequencing data or at least comment in a results section how did the sequencing perform.

Is the study design appropriate and does the work have academic merit?

Partly

Is the work clearly and accurately presented and does it cite the current literature?

Partly

If applicable, is the statistical analysis and its interpretation appropriate?

No

Are all the source data underlying the results available to ensure full reproducibility?

No

Are the conclusions drawn adequately supported by the results?

Partly

Are sufficient details of methods and analysis provided to allow replication by others?

Partly

Reviewer Expertise:

eDNA, metabarcoding, bioinformatics, nsDNA

I confirm that I have read this submission and believe that I have an appropriate level of expertise to state that I do not consider it to be of an acceptable scientific standard, for reasons outlined above.

# Response

**Published:** 2025-09-23

**Authors:** Nicolas Garcia Seyda

**Affiliations:** Institute of Research for Development, Marseille, France

We sincerely thank the reviewer for the time and effort dedicated to evaluating our manuscript, as well as for the constructive feedback provided. Below, we address each comment in a point-by-point manner:

 COMMENT 1: The authors sample 19 specimens from seven species of sponges from the same environment. One first drawback is that (methods section, first paragraph) "Samples of the same species were stored in Ziploc bags". In my opinion that gets rid of the value of replicates, and now you are left with seven samples. 

 RESPONSE 1: As now clarified in the main text, sample pooling is a common strategy in screening studies to reduce experimental costs and workload. Given the absence of prior knowledge on which species might perform better—and the possibility that some species might yield no detectable result (as occurred with three of the seven tested species)—biological replicates are initially pooled to generate a single sample per species. Once promising candidates are identified, a follow-up campaign with independent replicates is conducted to validate the initial findings. In recognition of the preliminary nature of these results, we have now added a “Limitations of the Study” section to explicitly highlight the constraints and drawbacks of this approach.

 COMMENT 2: The authors do their best for keeping extraction protocols standard across samples, although one can only imagine it is hard to standardize 0.5cm3 of tissue. 

 RESPONSE 2: The 0.5cm3 of tissue has been recurrently used in the literature (see Jeunen 2023, Cai 2022). In addition, DNA input was normalized following the extraction.

 COMMENT 3: The authors do a qPCR using fish-specific primers to estimate the amount of fish target DNA inside the sponges. Not mentioned in the main report, but the supplementary material states all samples are in a similar range of fish DNA concentration. The authors then treated the species with bigger numbers (no statistical test for differential concentration of fish DNA) differently to the rest, which they pooled in a single tube per sponge species. I think this is a bad choice. The sequencing results are not very promising, with only 6 fish species detected in 4 of the 7 sponge pools. This low diversity goes against the results shown in the qPCR assay, and all previous experiments on eDNA retention in sponges, suggesting something must have gone wrong on either the library preparation, the sequencing or the analysis. 

 RESPONSE 3: The inconsistencies between qPCR and sequencing results have now been clarified in the revised manuscript. Specifically, the underlying dataset, Methods, and Discussion have been updated to explain that the Fish16S primers likely produced artifactual amplifications. This is evidenced by the numerous MOTUs with a low best identity value (<0.65) in Table 6 (Underlying data), and may explain why *Chondrosia clathrus* and *Aplysina oroides* showed high apparent fish eDNA concentrations by qPCR, but yielded very few validated reads after bioinformatic filtering.

 Regarding sequencing performance, the results are in fact consistent with previous reports for Mediterranean sponges. Mariani et al. (2019) detected four and seven fish taxa from *Ircinia fasciculata* and *Petrosia ficiformis*, respectively, from two different sites and using 12S (Teleo02) primers. Corral-Lou et al. (2025) reported six fish taxa (four at species level) from 32 *Haliclona cinerea* and *Haliclona mediterranea* specimens sampled across four sites and two seasons with COI primers (mlCOIintF-XT / jgHCO2198). By comparison, our study based on 19 specimens collected at a single site during a single season recovered a comparable number of taxa.

 COMMENT 4: It is totally possible that Axinella spp. are better eDNA samplers than the other species collected here. However, the results shown are not enough to support or disregard this assumption. 

 I would encourage them to share the raw sequencing data or at least comment in a results section how did the sequencing perform.

 RESPONSE 4: The underlying dataset has been revised to include previously missing information, and the raw sequencing data has also been uploaded. In addition, the manuscript has been revised to adopt more cautious interpretations. Specifically, we have downgraded general claims regarding sponge performance and added a “Limitations of the Study” section to explicitly acknowledge the absence of statistical analyses, due to the screening nature of this study.

# Reviewer report: Reviewer response for version 1

**DOI:** 10.21956/openreseurope.21370.r52562

**Published:** 2025-04-18

**Authors:** Dannise V. Ruiz-Ramos

**Affiliations:** 1 University of Maryland Eastern Shore, Princess Anne, MD, USA

In this brief report, Garcia-Seyda et al. evaluate the usefulness of marine sponges as natural eDNA samplers for the Mediterranean Sea. They test seven sponge species based on their availability and microbial abundance (HMA vs. LMA). They conclude that *Axinella verrucosa* might be the best sponge to use as a natural eDNA sampler. It is unclear whether *A. verrucosa* is a common species, as only two individuals were found at the site. This is an interesting pilot study, but the limited data and low detections make recommending a specific species as a natural sampler challenging. From the limited data presented, I’m not sure if sponges would be a better alternative to water samples, but they could serve as an alternative for rapid monitoring during an emergency. 

 Specific comments: (There are no lines in the manuscript, so I tried to give approximate locations.)

 Last paragraph of methods: “Results indicated higher Fish16S detection per input DNA in the species *Clathrina clathrus a*nd *Agelas oroides*, which were therefore pinpointed as the best candidates and sequenced individually. The remaining samples were merged per species and sequenced as a pool.” There is no discussion on how this decision affected the results in Fig 2 and your conclusion. Were *A. verrucosa* samples pooled, while others were not? That could affect the diversity of MOTUs. This lack of information makes Figure 2 misleading.

 Methods: change “products were controlled on a 2% agarose gel” to “products were verified”

 Discussion:

 If *Clathrina clathrus* and *Agelas oroides *were the samples with higher 16S detections, why are there no results in Fig 2? I think this lack of sequencing results should be discussed. Why did you decide A. verrucosa performed better? There is no statistical test.

 Change: “Axinella verrucosa outperforming the rest by recovering five of the six detected fish species, thus emerging as the most promising candidate for biomonitoring applications” Change to 4 species?

 How many species from the genus *Diplodus* are found in the study area? If MOTU was only assigned to the genus level, it could be a degraded sequence of *D. sargus*. So, based on the MOTU information (no percent identity given), you have four species, not 6.

 Maybe as natural samplers the 3D structure of the sponge is more important than the species?

 Delete point in” standing 3D structure similar to A. verrucosa.,”

 Second paragraph: change “community” for species.

 “The detected fish community was composed of demersal and reef-associated species, highlighting the relevance of sponges as tools for benthic biodiversity monitoring. This is all the most relevant given recent findings suggesting that water eDNA is a poor proxy for benthic biodiversity, emphasizing the potential of sponges for capturing eDNA from cryptic and substrate-associated taxa.” Maybe the authors have data from other markers to sustain these claims, but based on the results here, sponges are also poor indicators of benthic biodiversity (only 2 benthic fish species were detected).

 “Overall, *A. verrucosa* stands out as a promising candidate for future eDNA sampling campaigns, yet protocol optimization and further testing of other LMA sponges is warranted.” I do not think the presented data supports this claim.

 Peer review form:

- Is the work clearly and accurately presented and does it cite the current literature? Yes
- Is the study design appropriate and does the work have academic merit? Yes, for a brief report. However, it is suggested that authors present the full data or adjust their conclusions.
- Are sufficient details of methods and analysis provided to allow replication by others? Methods are well detailed, analysis is deficient.
- If applicable, is the statistical analysis and its interpretation appropriate? Data is limited, but I understand it is a brief report. Please report the results of the negative PCR control. Were only two replicates run for the negative control? From Figure 2, samples were run in triplicate, but the qPCR full data in supplementary materials shows only one sample, and two PCR negative control. Check the MIQE guidelines for reporting qPCR data. Also, there are no statistical analyses to test whether high-microbial-activity sponges were different from low-microbial-activity sponges. The authors should be more cautious with their interpretation since only three samples had two or more positive PCR replicates, and one of those samples was a high-microbial-activity sponge. Based on Figure 2, A. verrucosa only recovered two more fish species than the other species.
- Are all the source data underlying the results available to ensure full reproducibility? No, the data is incomplete. The data from sample extraction to qPCR results is available. Supplemental data is unclear, authors need to include definition of columns and the metadata for the data to be useful. The metabarcoding sequence table and link to the sequence depository are missing (only the sequence count is available).
- Are the conclusions drawn adequately supported by the results? No. It is not clear if all the samples were sequenced; the sampling pooling tab suggests 16 samples were sequenced (MIOA001-04, MIOA007, MIOA010-14, MIOA16, MIO19), the table “MOTUs dataset per sample” shows a summary of counts for samples MIOA002, MIOA013, MIOA014, and MIOA019. Where are the rest of the samples? If they were not sequenced, please say so. It renders Fig2 misleading. Did the authors amplify and sequence *A. cavernicola*, *P. piriformis,* and *C. reniformis *and did not recover any sequence? If those samples were not amplified and sequenced, please remove them from Fig 2. 

Is the study design appropriate and does the work have academic merit?

Partly

Is the work clearly and accurately presented and does it cite the current literature?

Yes

If applicable, is the statistical analysis and its interpretation appropriate?

No

Are all the source data underlying the results available to ensure full reproducibility?

Partly

Are the conclusions drawn adequately supported by the results?

No

Are sufficient details of methods and analysis provided to allow replication by others?

Partly

Reviewer Expertise:

Marine biology, environmental DNA, environmental omics.

I confirm that I have read this submission and believe that I have an appropriate level of expertise to state that I do not consider it to be of an acceptable scientific standard, for reasons outlined above.

# Response

**Published:** 2025-09-23

**Authors:** Nicolas Garcia Seyda

**Affiliations:** Institute of Research for Development, Marseille, France

We sincerely thank the reviewer for the time and effort dedicated to evaluating our manuscript, as well as for the constructive feedback provided. Below, we address each comment in a point-by-point manner:

 COMMENT 1: “Results indicated higher Fish16S detection per input DNA in the species *Clathrina clathrus a*nd *Agelas oroides*, which were therefore pinpointed as the best candidates and sequenced individually. The remaining samples were merged per species and sequenced as a pool.” There is no discussion on how this decision affected the results in Fig 2 and your conclusion. Were *A. verrucosa* samples pooled, while others were not? That could affect the diversity of MOTUs. This lack of information makes Figure 2 misleading.

 RESPONSE 1: The main text and figure legend have been updated to clarify that all biological replicates were pooled, leading to 1 sequenced sample per sponge species, except for *Clathrina clathrus a*nd *Agelas oroides *which were sequenced individually but retrieved few sequences after bioinformatic analysis.

 COMMENT 2: change “products were controlled on a 2% agarose gel” to “products were verified”

 RESPONSE 2: The text has been changed accordingly.

 COMMENT 3: If *Clathrina clathrus* and *Agelas oroides *were the samples with higher 16S detections, why are there no results in Fig 2? I think this lack of sequencing results should be discussed. Why did you decide A. verrucosa performed better? There is no statistical test.

 RESPONSE 3: The discrepancy between qPCR results and retrieved sequences has been added to the main text and the underlying data. The figure and its legend have been updated to clarify the sample-pooling strategy.

 COMMENT 4: “Axinella verrucosa outperforming the rest by recovering five of the six detected fish species, thus emerging as the most promising candidate for biomonitoring applications” Change to 4 species?

 RESPONSE 4: We have updated to 5 taxa, as indeed some detections (Diplodus) did not arrive at species level. The general claim has also been downgraded to remain cautious about our conclusions.

 COMMENT 5: How many species from the genus *Diplodus* are found in the study area? If MOTU was only assigned to the genus level, it could be a degraded sequence of *D. sargus*. So, based on the MOTU information (no percent identity given), you have four species, not 6.

 RESPONSE 5: Several Diplodus species are found in the study area, including *Diplodus vulgaris*, *Diplodus sargus*, *Diplodus cervinus* and *Diplodus puntazzo*. Comprehensive MOTU data, including percent identity, have been added to the underlying dataset. MOTU “Fish16S_00009” (renamed *Diplodus* sp. 1) shows 98.5% similarity to *D. vulgaris* based on a manual BLAST search of GenBank records. However, the three *Diplodus* sequences retrieved are also 98.5% similar to each other, which appears to represent the average level of similarity within the genus. As a result, they cannot be confidently assigned to a particular species and were therefore reported at the highest reliable taxonomic resolution, namely the genus level (i.e., *Diplodus* sp.1, *Diplodus* sp.2).

 COMMENT 6: Maybe as natural samplers the 3D structure of the sponge is more important than the species?

 RESPONSE 6: Indeed, the impact of 3D structure on eDNA recovery is mentioned in the discussion. However, a quantitative morphometric analysis of the sampled specimens would be required to rigorously assess the relationship between structural complexity and eDNA yield.

 COMMENT 7: Delete point in” standing 3D structure similar to A. verrucosa.,”

 RESPONSE 7: The text has been changed accordingly.

 COMMENT 8: Second paragraph: change “community” for species.

 RESPONSE 8: The text has been changed accordingly.

 COMMENT 9: “The detected fish community was composed of demersal and reef-associated species, highlighting the relevance of sponges as tools for benthic biodiversity monitoring. This is all the most relevant given recent findings suggesting that water eDNA is a poor proxy for benthic biodiversity, emphasizing the potential of sponges for capturing eDNA from cryptic and substrate-associated taxa.” Maybe the authors have data from other markers to sustain these claims, but based on the results here, sponges are also poor indicators of benthic biodiversity (only 2 benthic fish species were detected).

 RESPONSE 9: The text has been clarified to state that sponges may be good indicators of substrate-associated or bottom-dwelling taxa, ie. species living in close proximity to the seabed. For fish, following Fishbase habitat-descriptors, this would refer to benthic, demersal and reef-associated species, in opposition to those thriving in the water column (strictly pelagic species).

 COMMENT 10: “Overall, *A. verrucosa* stands out as a promising candidate for future eDNA sampling campaigns, yet protocol optimization and further testing of other LMA sponges is warranted.” I do not think the presented data supports this claim.

 RESPONSE 10: The conclusion has been revised to adopt a more cautious interpretation: *“A. verrucosa appears to recover more fish eDNA, making it a promising candidate for further protocol refinement.”* Additionally, we have added a “Limitations of the Study” section to explicitly acknowledge the limitations of our work, including the inability to perform statistical analyses and, consequently, to definitively determine whether *A. verrucosa* or other LMA sponges are superior candidates for eDNA sampling.

 COMMENT 11: Is the study design appropriate and does the work have academic merit? Yes, for a brief report. However, it is suggested that authors present the full data or adjust their conclusions.

 RESPONSE 11: Claims have been downgraded and data completed to address the reviewer’s concerns

 COMMENT 12: Are sufficient details of methods and analysis provided to allow replication by others? Methods are well detailed, analysis is deficient.

 RESPONSE 12: Because of the screening nature of the project, and the subsequent pooling of samples, statistical analysis is not possible. This notion has been added to the main text.

 COMMENT 13: If applicable, is the statistical analysis and its interpretation appropriate? Data is limited, but I understand it is a brief report. Please report the results of the negative PCR control. Were only two replicates run for the negative control? From Figure 2, samples were run in triplicate, but the qPCR full data in supplementary materials shows only one sample, and two PCR negative control. Check the MIQE guidelines for reporting qPCR data. Also, there are no statistical analyses to test whether high-microbial-activity sponges were different from low-microbial-activity sponges. The authors should be more cautious with their interpretation since only three samples had two or more positive PCR replicates, and one of those samples was a high-microbial-activity sponge. Based on Figure 2, A. verrucosa only recovered two more fish species than the other species.

 RESPONSE 13: The legend of the figure has been updated to clarify the use of a total of eight PCR replicates, as described in the Methods. The underlying dataset has also been revised to include previously missing information, including the results of positive and negative PCR controls. Finally, the manuscript has been revised to adopt more cautious interpretations. Specifically, we have downgraded general claims regarding sponge performance and added a “Limitations of the Study” section to explicitly acknowledge the absence of statistical analyses, due to the screening nature of this study.

 COMMENT 14: Are all the source data underlying the results available to ensure full reproducibility? No, the data is incomplete. The data from sample extraction to qPCR results is available. Supplemental data is unclear, authors need to include definition of columns and the metadata for the data to be useful. The metabarcoding sequence table and link to the sequence depository are missing (only the sequence count is available).

 RESPONSE 14: The underlying data file has been revised to incorporate the corrections and address the reviewer’s concerns.

 COMMENT 15: Are the conclusions drawn adequately supported by the results? No. It is not clear if all the samples were sequenced; the sampling pooling tab suggests 16 samples were sequenced (MIOA001-04, MIOA007, MIOA010-14, MIOA16, MIO19), the table “MOTUs dataset per sample” shows a summary of counts for samples MIOA002, MIOA013, MIOA014, and MIOA019. Where are the rest of the samples? If they were not sequenced, please say so. It renders Fig2 misleading. Did the authors amplify and sequence *A. cavernicola*, *P. piriformis,* and *C. reniformis *and did not recover any sequence? If those samples were not amplified and sequenced, please remove them from Fig 2. 

 RESPONSE 15: All samples were sequenced, yet some did not retrieve any sequence after the bioinformatic cleaning steps. This has been now clarified in the methods and Fig 2 legend.
